# Unsettled grounds: religion, gender and sexual nationalism in a Dutch city

**DOI:** 10.1080/09637494.2024.2430096

**Published:** 2025-01-27

**Authors:** Brenda Bartelink

**Affiliations:** Faculty of Behavioural and Social Sciences, University of Groningen, Groningen, The Netherlands

**Keywords:** Secularity, material religion, sexual nationalism, urban ethnography

## Abstract

This contribution explores how the polarisation between religion, gender and sexuality in relation to nationalism manifests in the Dutch city of The Hague. Conceptual debates on secularism and sexual nationalism, as well as the materialist study of religion, are brought into conversation with ethnographic data on African Christian placemaking in the city. The contribution demonstrates how materialisations of Dutch secular nationalism become visible in religious spatial practices of religious migrant communities in their interaction with the city. It argues that African Dutch communities – youth in particular – experience and navigate the city as a space of multiplicity. They live, produce, and navigate contestations over gender and sexuality in secular and religious spaces in the city on a day-to-day basis. It offers alternative perspectives in relation to the political mobilisation of religion, secularity, gender and sexuality as culture wars or discursive battlefields in research and policy debates.

## Introduction

The English- and Twi-speaking community meets in an industrial hall decorated with colourful fabrics including one Dutch and one Ghanaian flag. When I enter the hall, it is filled with the sound of worship songs, accompanied by Ghanaian drums. This Sunday, a special youth-led church service takes place. It is opened by an adult speaker who talks about problems between youth and adults, and explores what obeying God means for the young generation. After that, three young people are invited to the pulpit to give their sermons. A young man responds: ‘Young people cannot come home and experience that parents listen to their emotions […]. We live in another world and parents do not understand that’. A young female adds later: ‘We learn to voice our opinions in school, yet when we come home to our parents it is expected that we obey them. Freedom of speech is our right, but our parents think we are too Dutch’. The preacher responds that young people can have their opinion, and obey their parents, because their parents are above them. In the pastor’s sermon on ‘Hearing and obeying God’ it is emphasised that youth should be aware of aspects of Dutch culture that do not fit with ‘our culture’, such as ‘acceptance of men marrying men or boys that want to change into girls’. (excerpt from field report 2018)

Freedom of speech and the right to make your own decisions regarding your body and sexuality have been at the heart of contestations around religion and secularity in contemporary postcolonial nation-states. In the Netherlands, this has often resulted in highly polarised debates in an increasingly secular progressive public domain (Schuh, Burchardt, and Wohlrab-Sahr 2012). The Netherlands is constructed as a liberal, secular progressive nation-state, expressed in claims of freedom of speech and women’s and gay rights as being typically Dutch (Balkenhol, Mepschen, and Duyvendak 2016). Minority religious and cultural communities are often portrayed as suffocating and hampering the freedom of young people, women, and sexual minorities (Bracke 2012). Muslims in particular, are framed as ‘backward’ and ‘violent’ in contrast to what is perceived as a ‘modern’ and ‘progressive’ Dutch nation, revealing an intersection of essentialist ideas on religion, culture, and race at play in distinguishing particular groups from the majority of society (Balkenhol, Mepschen, and Duyvendak 2016; M. De Koning 2020).

Against this background, it is both relevant and interesting to explore African Dutch Christian youth who actively bring their desires, questions, and explorations of freedom and rights into their cultural and religious communities. Such exploration suggests that their lives do not unfold in the binaries that characterise secular progressive discourse, but in the concrete material spaces of the city in which they live. By bringing their concerns about freedom and agency to the church, even unto the pulpit, they demonstrate that religious spaces are far from singular and can have multiple, diverging, and even conflicting meanings. This contribution draws on ethnographic research on African Dutch inhabitants of the city, to explore The Hague as a ‘frontier zone’ (Meyer 2018) in which religious and secular social worlds meet and interact within a material and spatial context. Contestations on religion and secularity that play out around gender and sexuality in particular, make visible such interactions.

In this contribution, I will think through ethnographic vignettes of African interfaith and Pentecostal spatial practices in The Hague. With the first vignette, I explore how ‘sexual nationalism’, e.g. the Dutch nation-state as an ‘imagined sexual community’ (Jaunait, Le Renard, and Marteu 2013, 4), is implied in the spatial practices of African Dutch Pentecostals in the city. In this case, the city of The Hague emerges as the aforementioned frontier zone. In subsequent sections I explore African Dutch youth’s experiences in navigating religion, gender and sexuality in the city and their parents’ understandings of and approaches to those experiences. These shed light on how contestations over religion, secularity, gender and sexuality are lived, produced, and navigated in city spaces. With this I aim to go beyond understanding the polarisation dynamics around religion, gender and sexuality as only a culture war or a discursive battlefield in contemporary nation-states.

### A materialist approach to religion, gender and nationalism in cities

This contribution is inspired by two key conceptual debates in the study of religion: 1) the study of sexuality, secularity and nationalism and 2) the spatial and materialist study of religion, and in particular of Pentecostal Christianity. That nationalisms are gendered and sexual is not new. In his classic analysis of sexual nationalism, George Mosse demonstrated that nationalism in Europe in the nineteenth century heavily relied on sexed and sexualised stereotypes (Mosse 2020). The seminal work by the historian Joan Scott suggested the term ‘sexularism’, which captures well how secularism has excluded both women and religion from the public domain since the onset of the Enlightenment (Scott 2017). Sexular nationalism, to combine both concepts, naturalised a gendered and unequal social order in which sexuality emerged as one of the primary domains through which contestations around religion and secularity take place (Cady and Fessenden 2013, 8).

Anthropologist Ann Stoler argues that the ‘history of Western sexuality must be located in the production of historical Others, in the broader force field of empire where the technologies of sex, self, and power were defined as “European” and “Western” as they were refracted and remade’ (Stoler 2002, 195). In her study of the Dutch colonial project in Indonesia, she demonstrates how the Dutch model citizen was produced through a rigorous organisation of intimate relations, embodied practices, and pedagogies in the families of the Dutch colonisers. While Dutch colonial sexuality was constructed with reference to Christianity, in postcolonial Netherlands technologies of sex and self draw on a secular version of progressive modernity (e.g. Keane 2007 on Christian and secular modernities; Bartelink and Wiering 2020).

It is important to note that the Netherlands has been seen as a multicultural model society since the 1970s (Duyvendak and Scholten 2011). Whilst there has not been a singular model of immigrant integration in the Netherlands since the 1970s, an assimilationist approach to immigrant integration has been promoted since the early 2000s (Duyvendak and Scholten 2011). Although the understanding of secularity for the sake of tolerance of religious differences that was characteristic of the Dutch system of pillarisation is still visible in legal and policy domains, public and political discourses have shifted towards secular progressiveness (Schuh, Burchardt, and Wohlrab-Sahr 2012). The construction of the Netherlands as a secular progressive nation works through contrasting the so-called liberal Dutch with their religious and cultural others, who are in turn seen as threatening Dutch progressive values regarding gender and sexuality (Balkenhol, Mepschen, and Duyvendak 2016; Bracke 2012). Dutch Muslims in particular have become a focus of racialised policies that (while seemingly neutral) aim to govern religion through distinguishing between ‘acceptable and unacceptable Islam’ (M. De Koning 2020). Yet, the secular progressive self-representation also materialises in policies and practices targeting broader immigrant and minority populations, such as citizenship rituals (Verkaaik 2009), pedagogical support programmes (van den Berg 2016), sexuality education in secondary schools (Wiering 2023), and sexual health programmes in cities such as Rotterdam, Amsterdam, and The Hague (Bartelink and Knibbe 2019). Finally, secular progressiveness is also observed in Dutch development programmes implemented in the Global South (Bartelink and Wiering 2020; Roodsaz and van Raemdonck 2018).

In light of this, African Pentecostal churches in the Netherlands can be seen as sites of action in which people, and men in particular, are (re-)made into morally responsible subjects who focus on stable family lives undergirded by moral and economic stability (Fesenmyer 2022). In particular, because migrants in the Netherlands are more likely to experience marital instability and teenage pregnancy (Caarls, Mazzucato, and Richou 2015), this moral responsibilisation may be an important strategy to realise family stability. Experiences of marginalisation and racialisation can strengthen the desire to find identity and belonging in the church. For African migrant and minority populations, their parenting identity is strengthened in church while contested in the public domain. Immigrant parents tend to be followed closely by authorities and institutions as their parenting can be seen as a potential threat to child wellbeing and development (A. De Koning and Ruijtenberg 2022; Maier and Coleman 2011; Okpokiri 2020). African Pentecostal churches in the Netherlands are sites of action that are located in the city, but also construct a particular attitude to the city (Maier and Coleman 2011). Churches often develop an active politics of educating and supporting their members to realise stable families, and to shield them from harmful interventions of the state – in that sense the youth service in the opening vignette is similar to the theatre performance that Maier and Coleman (2011) analyse in a Nigerian church in London, as efforts to navigate religious and state-related dilemmas around the rearing and moral socialisation of children.

Research such as Maier and Coleman’s fits with the material approach to the study of religion that has been taken up by scholars of Pentecostal Christianity (Garbin 2013, 2023; K. Knibbe 2009; Maier and Coleman 2011). This research draws attention to the importance of understanding how churches make up spaces of alterity at the margins of post-industrial European cities, in which confronting the ‘immorality of secular urban societies’ is key (Garbin 2013). This is not to suggest that secularity should be seen as un-African, but rather that it is situated differently in African contexts and may be observed in a more critical engagement with African Pentecostalism (van Dijk 2015). From an African Pentecostal perspective, controversies around gender and sexuality in the public domain are moral controversies. In this contribution, I will draw on the materialist study of religion (Vásquez 2011) to explore how these ‘occur in space’ and are lived and embodied in particular ways (Knott 2014, 126). In doing so, I aim to contribute not only to the materialist study of religion, but also to the materialist study of secularity and sexuality.

The ethnographic case studied in this contribution takes place in The Hague, a city with its own historical and contemporary dynamics of diversity distinct from those of Amsterdam or Rotterdam. The Hague is a city that presents itself and is internationally acknowledged as the ‘city of peace and justice’ (Hulleman and Govers 2011). It is home to international institutions such as the International Criminal Court and the Peace Palace, and various high profile NGOs such as Oxfam, Save the Children, and UNICEF. As a migration city, The Hague is highly politicised and prone to particular hierarchies that are highly visible in its material context as well as in local policies related to migration (e.g. Oomen and Leenders 2020). An expat migrant navigates between their white-collar job and home in an affluent neighbourhood on the western sea side of the city, while an inhabitant with a so-called refugee or migration background may move between a cleaning job in the same white-collar environment and a house in the Schilderswijk, the inner-city working class neighbourhood where 92% inhabitants are of non-European origin (Verloo 2018). Governance in The Hague is characterised by active nativist policies that create social and spatial divides between ‘native Dutch’ and ‘migrant’ inhabitants of the city seen as ‘living in different worlds’ (Hoekstra 2018). In 2014 a moral panic in the national media emerged around the alleged radicalisation of Muslim inhabitants of the Schilderswijk and Transvaal, which made visible that these social and spatial divides in the city intersect with religion (Rana 2022; Verloo 2018). Most notably, confrontations between Muslim youth and right-wing nationalist groups in July 2014 were amplified by media framing of the Schilderswijk as a ‘sharia triangle’ (Verloo 2018). Yet, much less attention was paid to the community initiatives to restore the safety of the neighbourhood (Verloo 2018), some of which were born out of interfaith networks that form an important part of the city’s social fabric. This demonstrates what Konyali, Haddad, and Pott (2019) observe in the German city of Osnabruck, that static ideals of tolerance and peace in The Hague do not allow for intersectional engagement with diversity.

## Methods

This contribution analyses data gathered through ethnographic fieldwork in The Hague between 2016 and 2019, focused on sexual wellbeing and religion in the city.^1^ The Hague is a culturally diverse city. Between 2006 and 2019 the proportion of inhabitants of non-European origin grew from 45% to 54.7% (Den Haag in Cijfers 2024). It is difficult to describe the specific demographics of the African Dutch population for the period of study, yet in 2022 the Central Bureau of Statistics reported that 28.9 out of 1,000 inhabitants of The Hague were born in a sub-Saharan African country (19.1) or born to parents that were born there (9.1) (CBS 2023). In addition, although their number is small in the Netherlands (289,000 inhabitants), sub-Saharan African migrants are considered a significant group in and around the city of The Hague (CBS 2023). I conducted fieldwork in ten churches, some Nigerian or Ghanaian, while others had a more diverse African attendance. Most of them were Pentecostal, while some were mainline denominations such as Catholic or Protestant churches with large African Dutch groups attending. In addition, I attended a year-long leadership programme in a Bible School connected to a large Nigerian transnational Pentecostal church. These churches are located in a Dutch city and therefore Dutch, however the leaders and the majority of their congregants were either born in societies on the African continent or raised by parents born there. In terms of their positioning in the Netherlands, these churches are attended by diverse groups of people, including those who were born in the Netherlands, those who have obtained citizenship and those who aspire to do so (K. E. Knibbe 2011). In the course of the fieldwork, I also interacted with policymakers, NGO professionals, public health experts, and cultural mediators (e.g. people from diverse African Dutch communities who voluntarily or professionally worked on improving access to public services). I conducted ten formal interviews, while most of my interviews were spontaneous. In addition, ten interviews with female youth in a Ghanaian Dutch Pentecostal church were conducted by Masters student Mirre van der Meer under my supervision, for a thesis project on bicultural youth. The interviews were conducted after verbal informed consent from the participants, formal interviews were recorded, while informal interviews and observations were documented in fieldnotes and subsequent field reports. In addition to participant observation and interviews, I gathered documents, including leaflets and books produced by churches and organisations that I engaged with during fieldwork. I also consulted websites and social media posts by the actors and groups I followed. As a participant observer I have always been transparent about my role as a researcher and the focus of my research, as I explain in more detail below.

While the theme of religion, gender and sexual nationalism – seen through the lens of parents’, youth and church leaders’ experiences and responses – came up at least once in most of the churches and interchurch gatherings I visited, this contribution explicitly engages with examples from two churches. One is the city branch of a large transnational Pentecostal church originating in Ghana, where Van der Meer conducted two months of fieldwork and which I visited four times throughout the fieldwork. The other examples come from my frequent interactions with a smaller, independent Pentecostal church, the pastor and the majority of the congregation of which had Nigerian origins. I visited the Sunday services regularly throughout my research, interviewed the pastor (who texted and phoned me regularly as well), interacted and spoke with church members during and after church, and was invited as a speaker and as a workshop leader during events such as International Women’s Day and the youth and parents meetings I discuss later in this contribution. In addition, I will include examples from meetings of interchurch gatherings and interfaith platforms, when they addressed themes related to sexuality, gender, youth, and parenting. These meetings were sometimes attended by pastors, yet more often by leaders of women’s and youth groups in the churches that I studied. I will also refer to observations and interviews conducted in other churches, where relevant to demonstrating how the themes addressed in this contribution emerged in multiple spaces during my fieldwork. The intention here is not to ethnographically study one particular church, like the scholars of Pentecostalism that I introduce in the literature review have often done, but to trace how African Dutch young people and their parents navigate the different and diverging secular and religious lifeworlds present in the city of The Hague.

When I embarked on fieldwork in September 2016, I had already been introduced to different lifeworlds in The Hague. I had lived in the city for two years between 2003 and 2005 for an internship and subsequent temporary employment at the Ministry of Foreign Affairs. In the years after, I was a frequent visitor of the city as a researcher and professional in the field of international development. The choice to do fieldwork in The Hague was informed by how at home I was in the city, even though I mainly knew the city as a highly educated professional navigating the world of international organisations. Cycling through the city to map the traces and spatial practices of my African Dutch interlocutors increased my awareness of the invisible boundaries that were a reality for many of them. Personal experience, in diverse European and African Christian contexts during my youth in evangelical Christian communities and as a researcher in Eastern Africa, aided participation in religious activities while embodying some familiarity (e.g. Bartelink 2022). Yet, I remained a visitor in the churches because of my whiteness, while often welcomed as an academic. For some this made my participation more valuable, while others challenged my privileges and positioning, which deepened my reflections (Bartelink 2022; Bartelink et al. 2020). The data that informs this contribution is based on convenience sampling, which means that those churches that allowed me access were part of the research. For some churches there was an obvious interest in converting white Dutch people to Christianity, while for some pastors my role as an academic in a religious studies faculty was interesting as they were – or aspired to be – engaged in academic training in religious studies themselves. The focus on sexual wellbeing in the broader study was experienced as challenging and invited critique by the churches I approached. Whilst understandable in relation to fears of being accused of sexism or discrimination in the media (e.g. K. E. Knibbe 2018), this limited my access to certain churches. Many of my interlocutors in the churches that were part of the fieldwork and in particular those mentioned explicitly had genuine concerns about issues related to sexuality and gender in the churches or broader communities, which enabled me to build rapport. The variety in interlocutors and materials allowed me to reach a point of saturation and test the validity of my observations.

## Results

### Dutch sexual nationalism in African Dutch Pentecostal religious practice

The youth service in the Ghanaian Dutch church introduced in the first vignette suggests that young people should be given more space to make their own decisions, while the theme of sexual diversity clearly demarcated the boundary between an acceptable space for choice and what should be rejected in Dutch society. In this section, I argue that rather than being simply an expression of religious conservative norms regarding sexuality, fears that young people are becoming too free must be seen against the background of parents’ understanding of the Netherlands as a secular progressive nation and how this manifests in their everyday life. I will demonstrate that this is not separate from but fully part of their everyday religious experiences. In order to explore this, I will draw on an ethnographic example of an evangelical Christian outreach to the red light district in The Hague held annually during a Pentecostal women’s conference organised by a group of female religious leaders from various African and Caribbean churches. These female religious leaders were all active participants in the interfaith gatherings I attended as part of my fieldwork, while one developed the church sex education programme that I discussed in an earlier publication, unpacking the gendered paradoxes visible in this case (Bartelink 2022). Here, I am interested in how and why the two areas in The Hague known as the red light district become a focus of Christian placemaking. More specifically, I understand it as an example that demonstrates that African Dutch adults (parents and religious leaders) experience the city as a frontier zone in which the concrete materialisation of a progressive secular approach to gender and sexuality poses all kinds of moral challenges for raising children in a Pentecostal and responsible manner. My exploration starts with a dream shared by the Nigerian-born and UK-based Pentecostal Pastor Miranda, who was annually invited by the female religious leaders to speak at the conference and lead the outreach.

In a chat with Pastor Miranda during the car ride from the conference hotel, the pastor recounts a dream she had before she first visited The Hague five years ago. In this dream, she saw herself standing before a tunnel from which a few women emerged. Miranda sings me the song she heard the women in her dream singing, repeating: ‘save the prostitutes’. ‘So I knew God was giving me an assignment, but I did not want to go’. She decided to share it with the female pastors in The Hague who organise the annual conference, and they told her: ‘God called you, Pastor Miranda, you have to go’. She describes how hard it was for her to go into the red light district, trembling and hesitant, experiencing that nobody wanted to talk to her. Yet, over the years, she experiences how the women open up, which she sees as God’s work in this area.

Experience of the active presence of God and of a divine calling is not uncommon in Pentecostal Christianity. The female pastors recognising the active presence of the divine in Pastor Miranda’s life demonstrate that individual religious experience shapes the social world of African Christian women in The Hague (e.g. Luhrmann 2005). Yet, what is particular about the place and space of the red light district in The Hague for it to appear in a divine dream? When I ask Pastor Miranda if she has been called to similar ministries in other cities where she leads women’s conferences, she replies that The Hague is the only city in which she combines preaching with evangelical outreach. What is important to note here is that the Netherlands, and its widely known progressive secular image as a country in which prostitution is legal and considered a tourist attraction, lives in a very real and experiential way in the dream and divine calling of this pastor. This, in turn, led to concrete practices in which the women practised ‘spiritual warfare’, not only by talking to sex workers about the gospel but also, and mainly, by walking through the streets in prayer. It was clear to me that this was not an easy task for these Christian women, and that the red light district was not merely a collection of city streets like those most of the women walk, cycle, or drive through on a daily basis. The moment of ‘entering’ the space was marked as significant through the prayers for protection and support that took place just a street away from the point at which we entered the space. The women reported feeling anxious and trembling, some of them shared thoughts of hesitance. After the outreach, intense prayer was needed to cleanse the bodies and minds of the Pentecostal women from the dark influences of the place. This demonstrates that interacting with the material space of the red light district is intense physical, mental, emotional and spiritual work.

The evangelical outreach demonstrates that the red light district is perceived, conceptualised and (re-)produced simultaneously as secular progressive and as a Pentecostal space. Whereas on a superficial level it speaks to the polarisation between secular and religious approaches to sexuality, considering the experience of the divine that initiated Pentecostal placemaking in a space primarily associated with secular progressiveness makes visible the multiplicity of space. The red light district is not only a space that is endowed with and invokes secular and religious meanings and interpretations, it is simultaneously local (The Hague red light district), national (Dutch secular progressive space), transnational (African Pentecostal space), and cosmic (a space where spiritual warfare takes place). It brings together multiple secular and religious social worlds and can be considered a frontier zone (Meyer 2018). The space of the red light district affords different emotions, practices, and meanings for the different people that use the space. The sex workers rejected the prayers of the women, because, as one of them said, ‘God should not be brought to this place’. This suggests we can consider the intention of the Pentecostal outreach to bring God to the red light district through spiritual warfare both confirmed and simultaneously criticised by the sex worker. In their outreach, the African Christian women not only denied the (religious) agency of sex workers, as I have argued elsewhere (Bartelink 2022), they simultaneously challenged and confirmed the dominant understanding of the Netherlands as a secular progressive nation.

### ‘We live in another world, and our parents do not understand that’

What do young African Dutch people mean when they say that their parents ‘live in a different world’? The youth and their parents live in the Netherlands, are inhabitants of The Hague, members of the same churches, part of the same transnational families, and are living together in the same households. Hence, they participate in the same spaces. To what extent is this experience of living in a different world influenced by how these spaces may afford different meanings to parents and young people? I will first introduce an ethnographic vignette from a church that organised a parent-youth encounter. I will unpack the vignette in three subsections: this one concerning children and young people’s experiences of parental concerns and discipline; a second subsection focusing on parents’ concerns and strategies in relation to how their children interact with and participate in the multiple social worlds in the city; and a third subsection giving an account of how frontier-zone tensions are mediated in churches and faith-based organisations.

In April 2019, I visited the independent Pentecostal church that meets every Sunday in a Christian school. I have visited this church several times for participant observation and for an interview with the Nigerian Dutch pastor. This time, the pastor is the one who invited me to a special service for parents and children, to facilitate a focus group for youth about their interactions with their parents. The majority of the group of young people, ranging between ten and 22 years old, seems eager to talk to me: ‘Our parents do not trust us’, says one of the older girls. ‘We cannot hang out with friends, we have to come home immediately after school, and we can never go to a party or sleepover because our parents fear that we drink alcohol, have boyfriends and experiment with sex. I have no intention of doing that, but I can never show that you can hang out and still hold onto Christian values, because they do not trust me’. One of the older female youths shared with me afterwards that she chose to study in a city far away from The Hague to experience some freedom to make her own choices.

This example from my fieldwork illustrates what I observed in other meetings and in the interviews conducted by Van der Meer – that young people feel under surveillance from their parents. Female interviewees, in particular, described how they were expected to come home immediately after school, and when they were late parents would call them repeatedly to find out where they were. In the home, they had to contribute to the household by cooking and doing chores. While there were similar high expectations of their school results, girls reported their brothers had fewer responsibilities in the household. Home and school are important spaces in the city in which young people spend their time, yet they report limited opportunities to navigate other social spaces. Young people shared their frustrations about not being allowed to attend a sleepover or party at a friend’s house. They emphasised that such social events were important to them, yet denied them for fear of their drinking alcohol and engaging in sexual relations. Some of them emphasised their frustration at not being trusted by their parents, as for them this was not about alcohol or sexuality but about enjoying a relaxed time with friends and classmates. Dating was also not allowed, unless it was clear that it was tailored towards an upcoming engagement and subsequent wedding.

The frustration of these youth often was that their parents did not trust them to make their own, morally sound, decisions. As a consequence they developed strategies to create a bit more space for themselves. Those that were themselves highly motivated to do well in school would take up volunteering tasks at school. Others would invent an extracurricular activity. This enabled them to hang out longer with their friends. The consequence of their experience of not being trusted was to do things secretly and hence confirm that they could not be trusted.

### Parenting in a secular progressive city

The focus group demonstrates what was observed across the multiple sites and events visited during the fieldwork, that parents are concerned about how children interact with and participate in places and activities in the city. Parents worry about the exposure of their children to liberal moralities in particular: ‘Our kids are exposed to “first world” culture’ said one parent during a meeting on parenting organised by a platform for international churches. Another example is the concern raised during an anonymous Q&A at a women’s conference, about a child that had come out as gay. A response by the pastor confirmed the reality that children will encounter sexual diversity ‘as they live in the twenty-first century’, and added that it is important for parents to teach children that the Bible considers homosexuality to be against nature. This responsibilisation of people, which has also been observed in wider contemporary Protestant discourses, both acknowledges and amplifies parental concerns (for responsibilisation cf. Burchardt 2018 on men in African Pentecostalism, Bochow, Kirsch, and van Dijk 2017 on Protestant ethics, and Ellis 2021 for a detailed conceptual and ethnographic account of responsibilisation in evangelical discourses in the United States).

At the same time, across the fieldwork sites parents mentioned – in one way or another – their struggle with a loss of influence and authority. One parent said during a meeting on youth ministry: ‘I was critical about how youth dress in church, but they tell me it is their culture and I can’t say everything [sic]’. Parents’ limited agency is often given meaning in spiritual terms. In the Bible School I attended, several classes discussed the dominance of cultural relativism in Dutch society as an explanation for this lack of authority, while at a Pentecostal women’s conference disobedient children and family conflicts were discussed as a ‘sign of the times’. The sign of the times here refers to the end times before the second coming of Christ (e.g. Ukah 2020), which is expected to bring all kinds of conflicts and disasters. A similar concern was visible in the Ghanaian Dutch church in the vignette, where the allegedly high suicide rates were discussed alongside concerns about sexual and gender diversity as a sign of the end times. Parents’ concerns about their children being impolite, or tempted to engage in immoral behaviour such as drinking alcohol or engaging in casual sexual relations, are therefore understood in spiritual terms. Parents’ experiences of having limited power and authority over their children are part of their experience of living in a frontier zone (Meyer 2018). Through the conceptual lens of the frontier zone, we can understand the city of The Hague as an in-between space in which different religious and secular approaches to gender and sexuality that are mutually contested are co-present. Parenting in such a context means having to navigate not only the co-existence of different moral orders, but also the experience of being seen as ‘backward’ […] ‘for failing to adopt modern, emancipatory views on, for instance, same-sex relations and gender diversity’ (Meyer 2018, 70).

Furthermore, the concept of frontier zone enables us to go beyond understanding parental loss of authority merely in moral or spiritual terms. Current perceptions of African Dutch Christians as ‘backward’ can be traced back to how colonial categories and standpoints have informed knowledge (Meyer 2018). This resonates with my observations of some parents who also understood their limited parental authority in terms of their marginalised and racialised positioning in the Netherlands. As a Nigerian Dutch mother put it during the aforementioned meeting on parenting organised by the platform for international churches, a child that is good for society because it is a good soccer player is considered Dutch. However, when it is rebellious it is ‘African, it is a child of black people’. She then introduced another challenge: ‘I want to send a good child to society, but if I discipline the child, they send the police. [This is] the society where they say you can do what you want, you actually can’t […] because you cannot discipline your children’. While the mother put it very clearly and eloquently, she was not the only parent voicing this. Experiences with surveillance, discrimination and racism and the fear thereof frequently came up in informal conversations with parents during the fieldwork (resonating with studies done by A. De Koning and Ruijtenberg 2022 in the Netherlands, Maier and Coleman 2011; Okpokiri 2020 in the UK). In these conversations, parents also expressed their struggles with refraining from physical discipline because it is illegal in the Netherlands. While their children are freer to make their own choices than parents want, parental strategies to influence their children are more limited. Parents find themselves in a catch-22 situation. Against the background of spiritual and racialised struggles that parents experience, their strategies to watch their children closely, demanding them home and giving them chores and tasks to do, can also be understood as ways to exercise authority and prevent the kind of immoral or bad behaviour they fear, while refraining from using physical discipline.

Coming back to the title of this section, that parents do not understand that their children live in another world, we could on one level conclude that there is a gap between young people’s everyday experiences and parents’ understandings and approaches to these experiences. However, while parents may not understand these lived experiences, on another level they seem to understand very well that their children live in a ‘different world’. Yet, parents are also concerned about the spiritual, social, and material challenges and risks their children are facing in these different social lifeworlds in which parents do not participate, or in which they experience their own marginalisation.

### Mediating frontier zone tensions

The young people we interacted with during fieldwork mentioned two spaces that were important to them in negotiating more space for making their own decisions: school and church.

The school is a paradoxical space; as outlined above, it gives way to all kinds of concerns. Yet, since parents often experience a significant loss in status after migration, schooling their children is an important strategy to upward mobility (Crul et al. 2017). One girl interviewed by Van der Meer explained that her parents were very strict when it came to attending school: ‘I have not come to the Netherlands so you can skip school and do what you want. If I could do what you are doing now, I would note down every word the teacher said’. Parents emphasise the importance of studying hard and doing well in school, and young people take that up as a strategy to negotiate more space for themselves, as demonstrated above.

In all the interviews with the adolescent girls in the Ghanaian Dutch church, the church as a space in the city was of particular importance. They mentioned the freedom it offered to hang out, make friends, and freely attend activities without constantly being watched by their parents. It is important to mention that churches are much more than spaces in which religious and institutional activities take place. Churches offer ample opportunity for more casual interaction, since weddings, births, birthdays, and occasions such as Mother’s Day are celebrated there. Such celebrations include special blessings or songs during the service, and often with food and cake after the church service, which gives plenty of space to youth to hang out in the corridors, chat and socialise. Churches are therefore not only religious spaces, but spaces for socialising and connecting with other people. The young people interviewed by Van der Meer (this study) also pointed out that the church was a space to connect them to their Ghanaian identity and community, in addition to having religious meanings.

In the context of the city of The Hague, the churches visited during the fieldwork and in particular those introduced explicitly in the methods section can be considered sites of action in which various discourses meet and interact. As indicated in the vignette at the start of the contribution, broader narratives of the Netherlands as a secular, liberal and immoral society are brought up in churches, to point out the dangers assumed for African Christians who could easily backslide from their Christian lifestyle (see also Garbin 2023). However, at the same time churches also demonstrate active practices to mediate between the different lifeworlds and to pragmatically respond to challenges that people in their congregations experience. A case in point here is the marriage service I attended in the Nigerian Dutch church where I facilitated the focus group with youth, of a couple that lived together and had several children. While this did not fit with the morals around relationships and marriage preached in church, only the words ‘blessing the marriage’ (instead of ‘consecrating the marriage’) in the liturgy booklet suggested that the full white wedding ceremony was not the start of the marriage as it is ideally envisioned. In interviews with religious leaders more examples were given of such practices in various churches. This suggests that churches are spaces in which strong moral discourses are combined with more pragmatic approaches to navigating differences.

In addition, churches and church leaders are sensitive to young people’s experiences of not being understood or of oppression by their parents. They play active roles in mediating tensions between young people and their parents. In interviews and interchurch gatherings, religious leaders reported their concerns with the behaviour of young people as well as parents struggling with how to parent that they observed in their communities. Motivated by this, religious leaders mediate in conflicts between parents and children and develop other mediating roles. One religious leader called on her congregation to support a teenage girl who was pregnant, while another taught sexuality education lessons in churches because she was aware of parents’ hesitance to bring up sexuality with youth. Notably, both these leaders were female.

The Youth Service and Parents and Children Service are examples of activities that religious leaders initiate within their churches to create space for young people to speak out and have a voice in the church. In the Parents and Children service this was combined with creating space for parents to voice their concerns. This focus group with parents, organised alongside the one I facilitated with the youth, was facilitated by a ‘chief’, someone recognised as a cultural leader in the African Dutch community. The outcomes of the children and the parents’ focus groups were reported back to a plenary, after which a conversation took place in which young people could actively voice their critiques on behalf of the group. Not having to speak directly to their own parents helped circumvent the age hierarchies that structure conversations between parents and children. The youth services that this contribution started with is another case-in-point. While space was offered for parents to voice their concerns about their children, when this turned into a rather harsh moral critique one of the leaders stepped up and intervened. Sharing that children and youth approached him as a pastor with experiences of violence, he told the parents that disciplining and critiquing children may also result in creating an unsafe environment in which children do not trust their parents and do not come to them with their problems. Finally, interchurch and interfaith meetings also organised activities to address such challenges, often offering concrete workshops to church and youth leaders on parenting bicultural children, on how to talk about morally challenging issues such as sexuality education as well as on how to identify and respond to violence in the home (see also Bartelink 2022).

All these examples speak to how the lives of the African Dutch youth do not unfold in discursive clashes that present them with either/or choices. Their experiences of parents ‘living in another world’ speaks to how they are raised in a frontier zone. Within this they actively seek to bring their desires, struggles, and questions into conversation, bringing these different worlds together, even when they clash. Their parents also navigate these differences in their everyday lives, while their parenting strategies reflect the responsibilities they feel for the spiritual, social, and material wellbeing of their children. As visible in the evangelisation in the red light district, navigating differences involves intense physical, emotional and spiritual labour, which African Dutch youth and their parents do on a daily basis. Churches play multiple and diverging roles. While the polarised dynamics between secular progressive and Christian moral frameworks can be observed here, churches can offer space for parents and youth to explore diverging moral frameworks without rejecting one or the other. This suggests that churches are spaces in which multiple and conflicting meanings do exist even though sexual nationalist discourses suggest otherwise. With this, I do not suggest that churches are liberal spaces in which young people are always accepted and do not have to fear judgement or oppression. African Dutch churches (like many other Christian and secular institutions for that matter) are structured by gender, class and age hierarchies. Churches are, however, spaces in which more diverse narratives and practices are visible than is often assumed from a secular perspective.

## Discussion and conclusion

### Growing (up) in the city

Religious diversity in The Hague cannot be seen outside of the powers that determine what it means to be a city of peace and justice. The outreach in the red light district makes clear that Dutch sexual nationalism lives in the imaginaries, experiences, and practices of religious *and* secular actors in the city. The evangelical outreach in the red light district made visible a ground that is unsettled, a frontier zone, that affords both secular progressive and African Christian engagement with the space. However, the rather polarised understanding of religion and secularity that is apparent in Dutch secular progressive and African Pentecostal discourse does not adequately reflect the paradoxes and complexities of being within the city. Through the lens of how African Dutch youth navigate the city, religious and secular formations appear to interact in a more ecological manner.

The contribution furthermore makes clear that these African Dutch youth and their parents navigate the challenges of living in such unsettled spaces on a daily basis, often with limited means of influencing dominant discourses and structures. African Dutch parents shape their parenting practices in the context of concerns about the negative spiritual influences their children are exposed to, and their responsibility to prevent moral liberalisation. At the same time they experience a loss of authority and agency to do so. The African Dutch youth in this contribution, while experiencing living in a different world than their parents, also actively seek to bring their desires, struggles, and questions into conversation. In doing so, in particular in church spaces, they bring different worlds together, even when they clash. Young people’s spatial practices are both interesting and significant. They challenge binary constructions of religion and sexuality that dominate secular progressive sexual nationalism in the Netherlands and reclaim the city as a religious and secular space in which their own gender and sexual lives unfold. This more ecological way of engaging with The Hague bears resemblance to the Muslim girls playing soccer researched by van den Bogert (2021), the kickboxing Muslim women Rana (2022) researched, as well as my research with the African Dutch women crafting Delft Blue (Bartelink et al. 2020). Engaging with the city, its neighbourhood and spaces, means experimenting with ways of living and being that grapple with its multiplicity.

Considering the engagement with the city by parents and youth as organic does not by any means suggest it is apolitical. Scholars of African American parenting in the USA have long argued that raising children in contexts of inequality and racism should be considered political work (hooks 2007). Kaulingfreks (2017) suggests in her work on the youth who protest in The Hague’s Schilderswijk that the voices of these youth should not be considered radicalised but as political in a context where they are denied a political voice.

In conclusion, this study suggests that a material and spatial approach offers a better understanding of how religion, gender, sexuality, and nationalism intersect and unfold in the everyday lives of African Dutch youth and their parents than research focused on the political mobilisation of gender and religion in national and international contexts. To that end, research on cities and neighbourhoods introduced here might be complemented by research in homes, families, and other relational networks, as it is within these contexts that living in diversity is practised before it is conceptualised.

## Note

1. At the time this study was conducted I was employed at the Faculty of Religion, Culture and Society at the University of Groningen, which did not require formal ethical approval to be sought for this type of research. All data has been anonymised and all interviewees gave verbal informed consent to be interviewed for the purposes of this research.
